# Low-level laser therapy regulates microglial function through Src-mediated signaling pathways: implications for neurodegenerative diseases

**DOI:** 10.1186/1742-2094-9-219

**Published:** 2012-09-18

**Authors:** Sheng Song, Feifan Zhou, Wei R Chen

**Affiliations:** 1 MOE Key Laboratory of Laser Life Science & Institute of Laser Life Science, College of Biophotonics, South China Normal University, No. 55 Zhongshan Avenue West, Guangzhou, Tianhe District, 510631, China; 2 Department of Engineering and Physics, University of Central Oklahoma, 100 North University Drive, Edmond, Oklahoma, 73034, USA

**Keywords:** Microglia, Inflammation, Phagocytosis, LLLT, TLR

## Abstract

**Background:**

Activated microglial cells are an important pathological component in brains of patients with neurodegenerative diseases. The purpose of this study was to investigate the effect of He-Ne (632.8 nm, 64.6 mW/cm^2^) low-level laser therapy (LLLT), a non-damaging physical therapy, on activated microglia, and the subsequent signaling events of LLLT-induced neuroprotective effects and phagocytic responses.

**Methods:**

To model microglial activation, we treated the microglial BV2 cells with lipopolysaccharide (LPS). For the LLLT-induced neuroprotective study, neuronal cells with activated microglial cells in a Transwell™ cell-culture system were used. For the phagocytosis study, fluorescence-labeled microspheres were added into the treated microglial cells to confirm the role of LLLT.

**Results:**

Our results showed that LLLT (20 J/cm^2^) could attenuate toll-like receptor (TLR)-mediated proinflammatory responses in microglia, characterized by down-regulation of proinflammatory cytokine expression and nitric oxide (NO) production. LLLT-triggered TLR signaling inhibition was achieved by activating tyrosine kinases Src and Syk, which led to MyD88 tyrosine phosphorylation, thus impairing MyD88-dependent proinflammatory signaling cascade. In addition, we found that Src activation could enhance Rac1 activity and F-actin accumulation that typify microglial phagocytic activity. We also found that Src/PI3K/Akt inhibitors prevented LLLT-stimulated Akt (Ser473 and Thr308) phosphorylation and blocked Rac1 activity and actin-based microglial phagocytosis, indicating the activation of Src/PI3K/Akt/Rac1 signaling pathway.

**Conclusions:**

The present study underlines the importance of Src in suppressing inflammation and enhancing microglial phagocytic function in activated microglia during LLLT stimulation. We have identified a new and important neuroprotective signaling pathway that consists of regulation of microglial phagocytosis and inflammation under LLLT treatment. Our research may provide a feasible therapeutic approach to control the progression of neurodegenerative diseases.

## Introduction

Microglia are considered to be central nervous system (CNS)-resident professional macrophages. They perform homoeostatic activity and mediate the innate defense system in the normal CNS. However, localized activation of microglia has been implicated in the pathogenesis of several neurodegenerative disorders, such as Parkinson’s disease (PD), Alzheimer’s disease (AD) and multiple sclerosis [1].

In the CNS, microglia can be activated directly by products of microorganisms, environmental toxicants, and protein aggregates like β-amyloid (Aβ) or indirectly after neurodegeneration is induced [2,3]. Once microglia are activated in neurodegenerating microenvironment, they have macrophage-like capabilities, including phagocytosis and production of inflammatory cytokine [4]. Activated microglia can phagocytose fibrillar Aβ (fAβ) or dead cells from the CNS and can secrete different neurotrophic factors for neuronal survival. However, once activated, they eventually become more detrimental by releasing proinflammatory molecules (nitric oxide (NO) and TNF-α), thus causing secondary damage to neurons and the surrounding cellular environment [5]. Therefore, activation of microglia has become a hallmark of neurodegeneration. It has been debated whether neuroinflammation is an underling cause or a resulting condition in neurodegenerative diseases.

Abundant proinflammatory cytokines and oxygen radicals are presented in AD and PD brains. In PD brains, the highest density of microglia can be found in the substantia nigra (SN). They are not only highly activated but are also highly clustered around dystrophic dopamine neurons, and neuromelanin pigment taken up from degenerated dopaminergic nerve cells is characteristically observed in SN phagocytes [3]. However, the resident microglia always fail to trigger an effective phagocytic response to clear Aβ deposits during AD progression [6]. Although it is indisputable that microglia-mediated neuroinflammation plays a key role in the pathogenesis of neurodegenerative diseases, the relationships between neurotoxicity and phagocytic function of the activated microglia remain unclear.

Toll-like receptors (TLRs) are a class of conserved receptors that serve as an important link between innate and adaptive immunity. Microglia possess a number of TLRs, which play an important role in microglial activation in the brain of individuals with PD and AD. Recent studies indicated that peripheral lipopolysaccharide (LPS) injection caused microglia-related over-expression of TLR2 in aged mice [7], and that fAβ peptides activate microglia via TLR2 signaling pathway [8]. Other results show that the levels of TLR4 messenger RNA (mRNA) are upregulated in APP transgenic mice [9], and that the upregulation of cytokines is TLR4 dependent in an AD mouse model [10]. Targeting TLRs may be an important step to attenuate microglial activation in the CNS during AD or PD pathology.

Low-level laser therapy (LLLT) can modulate a broad-spectrum of cellular processes ranging from proliferation to apoptosis. It has been reported that the effects of laser irradiation on cell proliferation or inhibition are related to light fluence [11,12]. This phenomenon of photobiomodulation has also been widely applied in the treatment of skeletal muscle regeneration [13], wound healing [14], and skin wound care [15]. Studies have shown that Aβ-induced cell apoptosis was significantly diminished with light irradiation [16]. Furthermore, it has been demonstrated that LLLT has preventive effects on Aβ_25-35_-induced cell apoptosis, in which LLLT promoted YAP cytoplasmic translocation and inhibited Aβ(25–35)-induced YAP nuclear translocation [17].

Src kinases, non-receptor tyrosine kinases, are activated by oxidative events [18]. They are critically involved in fundamental cellular processes, including cell proliferation, migration and phagocytosis [19]. Recently, Han *et al.* observed that CD11b negatively regulated TLR-triggered inflammatory responses in macrophage by increasing Cbl-b-mediated degradation of MyD88 and TRIF, which depended on Src-Syk activation [20]. The involvement of LLLT-induced Src activation at relatively high laser doses in cells has been identified [21]. Since microglia can be beneficial by phagocytosing Aβ or harmful by secretion of neurotoxins, we hypothesized that Src may be involved in microglial functional regulation under LLLT. Thus, the effect of LLLT on microglia functions needs to be clarified in developing strategies to slow or prevent the progression of AD or other inflammation-mediated neurodegenerative diseases.

In this study, we investigated the effects of LLLT on LPS-activated microglia-induced neurotoxicity using microglia-like BV-2 cells and neuron-like neuroblastoma SH-SY5Y cells. We also examined the phagocytic effects of microglia and the interaction between microglial phagocytosis and neuroinflammation during LLLT. Our results suggested that LLLT could induce Src activation for neuroprotection by attenuating microglia-mediated inflammation and by enhancing microglial phagocytic activity.

## Materials and methods

### Chemicals and plasmids

The following reagents were used: LPS purified from *Salmonella typhimurium* (Sigma-Aldrich, St. Louis, MO, USA) to stimulate microglia; wortmannin and LY290042 (Sigma-Aldrich) to inhibit phosphatidylinositol 3-kinase (PI3K); API-2 (Sigma-Aldrich) to inhibit Akt; SMT ((C_2_H_6_N_2_S)_2_Â·H_2_SO_4_) (Sigma-Aldrich) to inhibit iNOS; PTIO (Beyotime Biotech., Haimen, Jiangsu, China) to scavenge NO; FITC-phalloidin (Sigma-Aldrich) to stain F-actin; latex beads (Sigma-Aldrich) to detect microglial phagocytosis; and propidium iodide (PI), CFSE, PKH26 and PKH67 (Sigma-Aldrich) to stain cells. Dual Luciferase Reporter Gene Assay kits were purchased from Beyotime Biotechnology and Nuclear/Cytosol Fractionation Kits were purchased from Biovision (Cambridge BioScience, Cambridge, U.K.).

The following antibodies were used: rabbit anti-MyD88, rabbit anti-GAPDH, rat-anti-Histone 3, rabbit anti-iNOS, and rat anti-β-actin. All were purchased from Santa Cruz Biotechnology (Santa Cruz, CA, USA). Antibodies specific for phosphorylated Syk Y519/520, Src Y416, FAK Y397, FAK Y861, Akt ser473, and Akt Thr308 were all purchased from Cell Signaling Technology (Beverly, MS, USA). Rabbit anti-Akt, mouse anti-FAK and rabbit anti-Rac1 antibody were obtained from Santa Cruz Biotechnology.

In addition, we used jetPEI™-macrophage transfecting reagent (Invitrogen, Carlsbad, CA, USA) to transfect plasmid DNA into cells and the cells were examined 36 to 48 h after transfection. The plasmid of pRaichu-Rac1 was kindly supplied by Dr. Michiyuki Matsuda. Rac1Q61L, Rac1T17N and wt Rac1 were purchased from Upstate Biotechnology (Lake Placid, NY, USA). Dr. Dianne Cox kindly provided the shRNA Syk and scramble shRNA. GFP-FRNK was a gift from Dr. Thomas Parsons. Dr. X. Shen (Institute of Biophysics, Chinese Academy of Sciences) kindly provided the pNF-κB-Luc. Dr. David A. Geller kindly provided the iNOS-Luc, and the pRL-TK was purchased from Promega (Mannheim, Germany).

### Cell culture

Murine microglia-like cell line BV-2 was maintained in Dulbecco’s modified Eagle’s medium (DMEM) with 15% heat-inactivated fetal calf serum (FCS), penicillin (100 units/ml), and streptomycin (100 μg/ml) in 5% CO_2_, 95% air at 37°C in a humidified incubator. To generate activated microglia, we stimulated cells LPS alone (100 ng/ml) or with different concentrations of inhibitors before LLLT treatment.

Primary microglia were isolated from postnatal day 1 to day 2 mouse brains (C57BL/6 J), as described previously [22]. Cells were cultured in DMEM/F12, with 20% fetal bovine serum (FBS), penicillin (100 units/ml), and streptomycin (100 μg/ml) in 5% CO_2_, 95% air at 37°C in a humidified incubator. Astrocytes were separated from the microglial cultures using a mild trypsinization protocol described by Saura *et al.*[23].

The present study was performed in accordance with the guidelines of the Guide for the Care and Use of Laboratory Animals (Institute of Laboratory Animal Resources, Commission on Life Sciences, National Research Council. Washington, DC: National Academy Press, 1996.); it was approved by the Institutional Animal Care and Use Committee of our university (South China Normal University, Guangzhou, China). The human neuroblastoma cell line SH-SY5Y was cultured in DMEM containing 10% heat-inactivated FBS, penicillin (100 units/ml), and streptomycin (100 μg/ml) in 5% CO_2_, 95% air at 37°C in a humidified incubator.

### LLLT treatment

The experiment was conducted as described in our previous work [24]. After 36 to 48 h of serum starved with 0.5% FBS, the microglial cells were irradiated with He-Ne laser (632.8 nm, 64.6 mW/cm^2^, HN-1000), (Laser Technology Application Research Institute Co., Ltd., Guangzhou, China) for 0.8, 1.33, 2.66, 5.32, 6.66, or 13.33 min in the dark, with the corresponding fluences of 3, 5, 10, 20, 25, and 50 J/cm^2^ respectively.

### Transient transfection and luciferase activity

Transient transfection of cells was performed using jetPEI™-macrophage transfecting reagent according to manufacturer’s instructions (Polyplus-transfection, Strasbourg, France). For luciferase activity assay, microglial cells in a 48-well plate (5 × 10^4^ cells/well) were incubated with plasmids containing 0.3 μg of pNF-κB or iNOS reporter luciferase plasmid. We used pRL-TK as an internal control of transfection efficiency. To correct for differences in transfection efficiency, each group of cells was transfected with 30 ng of pRL-TK and incubated overnight. The ratio of luciferase activity to pRL-TK activity in each sample served as a measure of normalized luciferase activity. Forty-eight hours after co-transfection, the cells were incubated with 100 ng/ml LPS for 12 h, and then cells were treated with LLLT and cultured for another 6 h. Cell extracts were prepared for determination of luciferase activity using Dual Luciferase Reporter Gene Assay Kit according to the manufacturer’s instructions. Luciferase assays were performed on a 96-well plate reader (Tecan Infinite M200, Tecan Group Ltd, Mannedorf, Switzerland) for 20 s. Results are expressed as the ratio of luciferase to pRL-TK (mean ± SEM).

### Confocal laser scanning microscopy (LSM)

Fluorescent emission from FITC was monitored confocally using a commercial laser scanning microscope (LSM 510/ConfoCor2) combination system (Zeiss, Jena, Germany) equipped with a Plan-Neofluar 40×/1.3 NA Oil DIC objective. FITC fluorescence was excited at 488 nm with the Ar-Ion laser (reflected by a beam splitter HFT 488 nm), and the fluorescence emission was recorded through a 500 to 530 nm IR band-pass filter. For intracellular measurements, the desired measurement position was chosen in the LSM image. To quantify the results, the average emission intensities from desired measurement positions were processed with Zeiss Rel3.2 image processing software (Zeiss, Jena, Germany).

### Nitric oxide measurement

Nitric Oxide (NO) production in microglia was detected with the fluorescent probe DAF-FM DA. DAF-FM DA is a cell permeable fluorescent probe for the detection of NO. It can passively diffuse across cellular membranes; once inside cells, it is deacetylated by intracellular esterases to become DAF-FM [25]. DAF-FM is essentially non-fluorescent until it reacts with NO. With excitation/emission maxima of 495/515 nm, the fluorescent intensity and images of DAF-FM can be detected by a 96-well plate reader and by confocal laser microscopy, respectively.

### Western blot analysis

Expressions of proteins were quantified by western blot analysis. After individual incubations, cell proteins were extracted in lysis buffer (50 mM Tris–HCl pH 8.0, 150 mM NaCl, 1% TritonX-100, 100 μg/ml PMSF) supplemented with protease inhibitor cocktail set I for 60 min on ice. After centrifugation (4°C, 12,000 rpm, 20 min), the resulting lysates were resolved on 8% SDS-PAGE Bis-Tris gels (30 mg/lane; Invitrogen, Life Technologies, Grand Island, NY, USA) and transferred to nitrocellulose membranes (Millipore, Bedford, MA, USA). The membranes were blocked in TBST (10 mM Tris–HCl, pH 7.4, 150 mM NaCl, 0.1% Tween-20) containing 5% non-fat milk and then incubated with a designated primary antibody and a secondary antibody. The signals were detected with an ODYSSEY™ Infrared Imaging System (Li-Cor, Lincoln, NE, USA). The intensity of the western blot signals was quantitated using ImageJ software (NIH, Bethesda, MD, USA), and the densitometry analyses are presented as the ratio of protein/β-actin protein, and are compared with controls and normalized to 1.

### Rac1 activity assay

We measured Rac1 activity in LLLT treated microglial cell lysate using Rac1 Activation Assay Kit (Upstate Biotechnology, Lake Placid, NY, USA) according to the manufacturer’s instructions.

### Microglia cytotoxicity assay

Cytotoxicity of microglial cells was assessed using a Transwell™ cell-culture system or a coincubation system. In the Transwell™ cell-culture system, 5 × 10^6^ microglial cells in 1 ml medium were added to the upper chamber of 6-well Transwell™ plates with 0.4-μm pores (Costar, Corning Inc., NY, USA), and cells were incubated with 100 ng/ml LPS for 10 h at 37°C. After being washed with PBS, target SH-SY5Y cells were resuspended at 1 × 10^6^ cells/mL and labeled with 1 mM carboxyfluorescein diacetate succinimidyl ester (CFSE) (Molecular Probes Europe, Leiden, The Netherlands) for 8 min at 37°C. The labeling reaction was quenched by the addition of cold DMEM containing 10% FBS. After washing with PBS, the CFSE-labeled target cells in 1.5 ml medium were added to the lower chamber of Transwell™ plates. When microglia were exposed to light, we took the upper chamber in a new 6-well plate for LLLT. Immediately thereafter, the 6-well plate was replaced by the lower Transwell™ chamber with SH-SY5Y cells to microglial cells at a ratio of 1:8 at 37°C in a humidified atmosphere of 5% CO_2_. By using this procedure, we prevented the direct impact of LLLT on the neuronal cells. In co-culture conditions, microglial cells were treated with LLLT. After 24 h incubation, cells in the lower chamber were harvested, propidium iodide (PI) was added to stain dead cells, and all samples were directly analyzed by fluorescence-activated cell sorting (FACS). Target cells killed by microglial cells were represented by the cell population showing double-positive staining for CFSE and PI.

In a coincubation system, 5 × 10^6^ PKH26 (red)-labeled microglial cells with 100 ng/ml LPS in 1 ml medium were added to the 6-well plate. After 10 h incubation, the cells were treated with LLLT. Then 1 × 10^6^ PKH67 (green)-labeled SH-SY5Y cells in 1.5 ml medium were added to the treated microglial cells. After 24 h coincubation, mix-cultured cells were harvested and PI was added to stain dead cells. All samples were analyzed by FACS. Target cells killed by microglial cells were represented by the cell population showing double-positive staining for PKH67 and PI/PKH26. Based on the evidence that phagocytes engulf dead cell corpses but not living cells, the dead PKH67-labled SH-SY5Y cells phagocytosed by PKH26-labled microglia can also be represented by the cell population showing double-positive staining for PKH67 and PKH26/PI.

### Phagocytosis assay

Microglial cells were collected and 1 × 10^5^ cells were cultured in 35 mm glass-bottomed dishes overnight. The cells were incubated in the presence or absence of the inhibitors, and then subjected to LLLT treatment. The fluorescent microspheres, as a marker of phagocytosis, were added to the treated cells at indicated time points after having been washed in PBS containing 0.1% BSA. Cells were then fixed with 4% paraformaldehyde, and three random fields of cells (>100 cells) were counted under a confocal microscope.

Phagocytic efficiency was determined by using the method of Pan *et al.*[26]. Briefly, the phagocytic efficiency was based on a weighted average of ingested microspheres per cell. The number of cells containing microspheres, the number of microspheres per cell, and the total number of cells were counted respectively. Phagocytic efficiency was calculated:

(1) $$\;\left(\%\right)=\left(1\times \text{X}1+2\times X2+3\times \text{X}3.+\text{n}\times \text{Xn}\right)/\left(\text{total number of cells}\right)\times 100\%$$

where Xn represents the number of cells containing n microspheres (n = 1, 2, 3, up to a maximum of 6 points for more than 5 microspheres ingested per cell).

### Phalloidin staining

The treated cells were rinsed with PBS before being fixed in 3.7% formaldehyde and washed again in PBS. The cells were then incubated at room temperature with 0.1% Triton X-100 buffer for 5 min and washed again in PBS. FITC-phalloidin (1:50 diluted in PBS) was added to the cover slips and incubated at room temperature protected from light for 30 min. The cover slips were mounted on glass slides. The FITC-labeled phalloidin was viewed under a confocal microscope.

### Quantitative measurement of F-actin

To assess the total F-actin content in microglial cells, microglia were treated with LPS for indicated time periods. The reaction was stopped by the addition of formaldehyde (3.7% final, V/V) for 15 min at room temperature. The fixed cells were then permeabilized with 10 mM imidazole, 40 mM KCl, 10 mM EGTA (ethylene glycol tetraacetic acid), 1 mM MgCl2, and 1% Triton X-100 at 4°C for 15 min. F-actin was then stained with FITC-phalloidin (Molecular Probes™, Life Technologies, Grand Lake, NY, USA) for 2 h at room temperature. After the cells were washed with PBS, F-actin-bound FITC-phalloidin was extracted with methanol. The extracts were centrifuged to remove any insoluble material, and relative fluorescence was measured using a 96-well plate reader with excitation and emission wavelengths set at 465 and 535 nm, respectively. The F-actin ratio was calculated as:

(2) $$\left(\text{F}-\text{actin in treated cells}-\text{background}\right)/\left(\text{F}-\text{actin in indicated cells}\;\left(\text{expression wild}-\text{type Rac1 cells in Figure 5B or untreated}\text{ cells in Figure 6E) - background)}\right)-\text{background}\right)$$

### Statistical analysis

Data are from one representative experiment among at least three independent experiments and are expressed as the mean ± SEM. Significant differences between groups were compared using the one-way ANOVA procedure followed by Student’s *t* tests using SPSS software (SPSS Inc., Chicago, IL, USA) and the differences were considered statistically significant at *P* <0.05.

## Results

### Effects of LLLT on LPS-activated microglia-induced cytotoxicity

**Figure 1:**
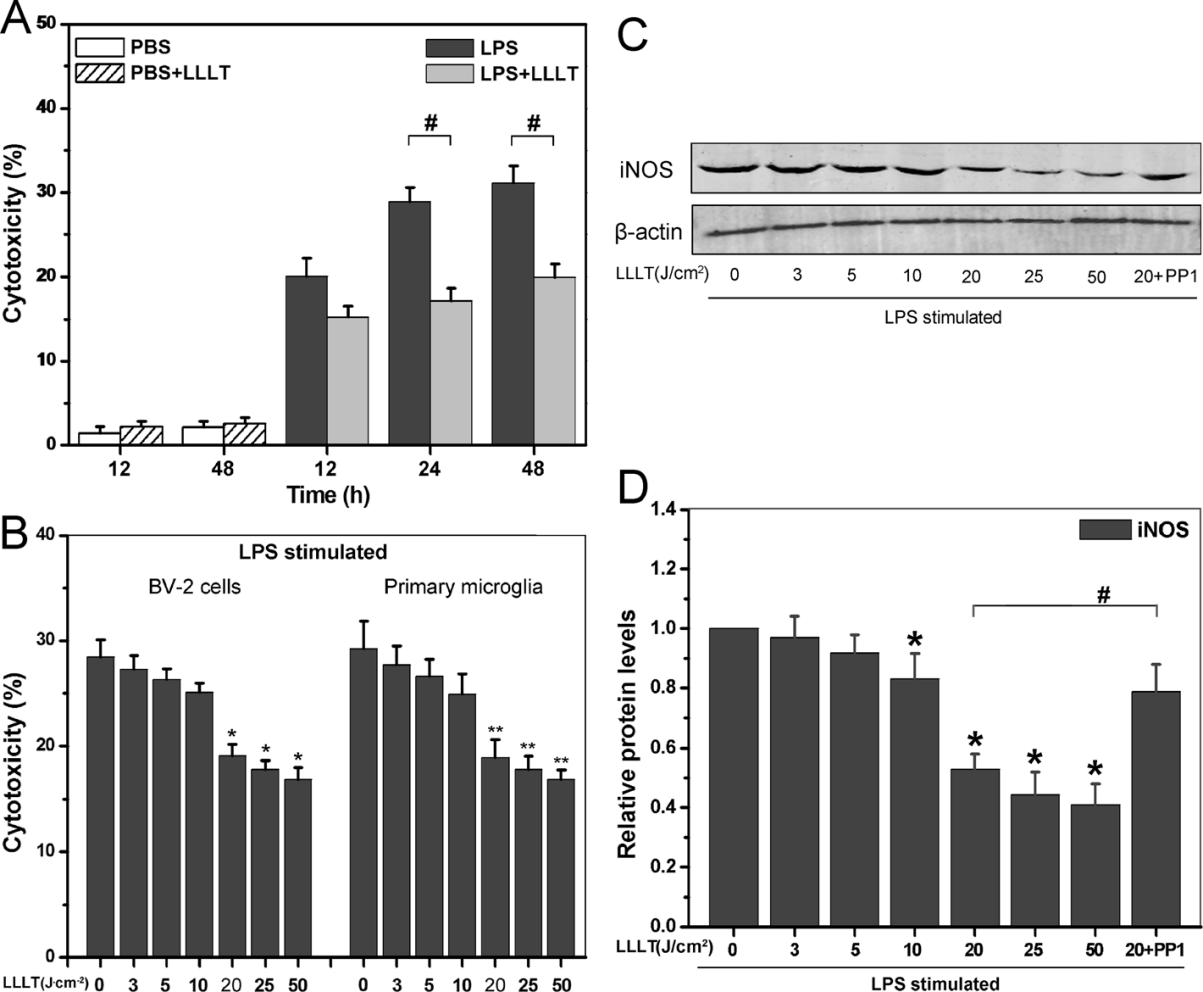
**Effects of LLLT on LPS-activated microglial cells.** SH-SY5Y cells were cultured (**A**) in the presence of LLLT-treated (20 J/cm^2^) LPS-activated BV-2 cells for 12, 24, and 48 h or (**B**) in the presence of the indicated doses of LLLT-treated BV-2 cells or primary microglia for 24 h. (A and B) Effector cells (microglia) were incubated with target cells (SH-SY5Y) at an E:T ratio of 8:1. After incubation, target cells that were double positive for CFSE and PI were analyzed by flow cytometry (n = 4; *P <0.05 and **P <0.05 *versus* corresponding control cells, #*P* <0.05 *versus* indicated cells). (**C**) BV-2 cells pretreated with or without PP1, then subjected to LLLT treatment, followed by western blot analysis of LPS-activated BV-2 cells received different doses of LLLT treatments to detect protein levels of iNOS. (**D**) Quantitative analysis of iNOS protein levels in treated cells. Data represent mean ± SEM (n = 4; *P <0.05 *versus* control cells, #*P* <0.05 *versus* indicated cells). CFSE, carboxyfluorescein diacetate succinimidyl ester; iNOS, inducible nitric oxide synthase; LLLT, low-level laser therapy; LPS, lipopolysaccharide; PI, propidium iodide.

To examine the anti-inflammatory effect of LLLT on LPS-activated microglia, we used neuronal cell line SH-SY5Y, which is an in vitro model to mimic responses of neurons. As shown in Figure 1A, cell death was attenuated by treatment of LLLT (20 J/cm^2^), and the relative death rate (LPS treated cells - LPS + LLLT treated cells) was lowered by LLLT to a similar extent at both 24 h and 48 h. Therefore, we used 24 h as the optimal time in the following microglial cytotoxicity experiments.

To determine the optimal dose of LLLT, LPS-activated BV-2 cells or primary microglia were treated by LLLT of different doses (3, 5, 10, 20, 25 or 50 J/cm^2^) and co-cultured with SH-SY5Y cells for 24 h. Microglia-mediated cytotoxicity was evidently reduced by LLLT in a dose-dependent manner (Figure 1B). While inducing the lowest microglia-mediated neurotoxicity, laser irradiation with a dose of 25 or 50 J/cm^2^ caused a slight contraction of microglial cells in some cases (data not shown), probably due to the toxic effect of laser irradiation with high doses. Therefore, unless stated otherwise, we used 20 J/cm^2^ as the optimal dose in the following experiments.

Since LPS led to abundant NO and inducible NO synthase (iNOS) induction in microglia, next iNOS was used to examine whether LLLT are capable of attenuating such proinflammatory molecule in microglia. As expected, LLLT inhibited the iNOS protein expression in a dose-dependent manner (Figure 1C, D). NO accumulation in BV-2 cells was also observed under LLLT using fluorescent probe DAF-FM DA (data not shown).

### LLLT-mediated neuroprotection requires Syk-dependent degradation of MyD88

**Figure 2:**
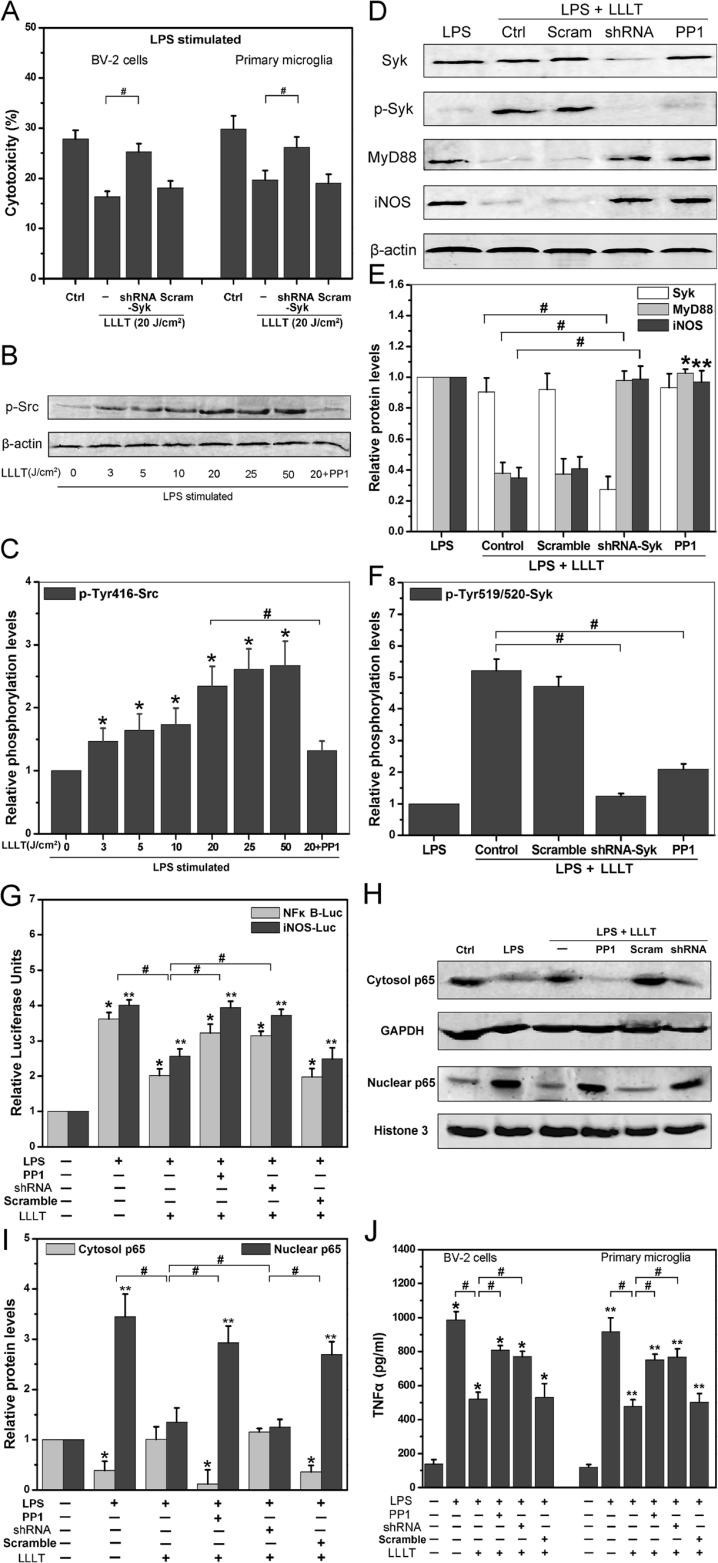
**LLLT-mediated neuroprotective effects dependent on Src/Syk-induced TLRs signal inhibition.** (**A**) Effects of Syk on microglial-induced cytotoxicity after LLLT (20 J/cm^2^) treatment. (n = 4; *P <0.05 and **P <0.05 *versus* corresponding control cells, #*P* <0.05 *versus* indicated cells). (**B**) Representative western blot analysis of LPS-activated BV-2 cells (pretreated with or without 10 μM PP1) received different doses of LLLT treatments to detect phospho-Tyr416-Src. (**C**) Quantitative analysis of the levels of phosphor-Tyr416-Src in treated cells. Data represent mean ± SEM (n = 4; *P <0.05 *versus* control cells, #*P* <0.05 *versus* indicated cells). (**D-F**) Representative western blot and quantitative analysis of total Syk, phospho-Tyr519/520-Syk, total MyD88, and total iNOS in treated cells. Data represent mean ± SEM (n = 4; *P <0.05 and **P <0.05 *versus* corresponding control cells, #*P* <0.05 *versus* indicated cells). (**G**) Relative NF-κB and iNOS luciferase activities in primary microglia after LLLT treatment. Data represent mean ± SEM (n = 6; *P <0.05 and **P <0.05 *versus* corresponding control cells, #*P* <0.05 *versus* indicated cells). (**H**)Representative western blot analysis of p65 in cytoplasmic (Cytosol) and nuclear fractions from lysates of BV-2 cells after LLLT treatment. (**I**) Quantitative analysis of p65 protein levels in treated cells. Data represent mean ± SEM (n = 4; *P <0.05 and **P <0.05 *versus* corresponding control cells, #*P* <0.05 *versus* indicated cells). (**J**) TNF-α secretion by microglia under different treatments. Data represent mean ± SEM (n = 6; *P <0.05 and **P <0.05 *versus* corresponding control cells, #*P* <0.05 *versus* indicated cells). iNos, inducible nitric oxide synthase; LLLT, low-level laser therapy; LPS, lipopolysaccharide; TLR, toll-like receptor.

Next we explored possible signal-transduction pathways in attenuating proinflammation response in LPS-activated microglia after LLLT treatment. Until recently, most studies suggested that Src and Syk tyrosine kinases were important in regulating TLR signaling [20,27]. Therefore, we adopted the RNA interference (RNAi) technique to investigate the role of Syk in TLR-mediated inflammatory response in microglia under LLLT. Knockdown of Syk by shRNA significantly suppressed LLLT-mediated microglial neuroprotective effects, indicating that Syk activation contributed to the TLR-targeted inflammatory response (Figure 2A).

One important finding by Han and colleagues was that CD11b-mediated anti-inflammatory response was dependent on Src-mediated Syk activation by dephosphorylating TLRs and their adaptors [20]. To determine whether LLLT could inhibit LPS-activated, microglia-induced cell death due to Src activation, LPS-activated BV-2 cells were treated with LLLT (3, 5, 10, 20, 25 or 50 J/cm^2^). The phosphorylation level of Src at Tyr416, which was indicative of Src activation, was evidently increased at 5 min by LLLT in a dose-dependent manner (Figure 2B, C). Pretreatment of cells with PP1, a specific inhibitor of Src kinase, significantly inhibited the activation of Src and iNOS expression by various doses of LLLT (as shown in Figure 1C and D).

To determine whether LLLT-mediated neuroprotection is required for Syk-mediated adaptors degradation, we examined the expression of MyD88 and iNOS using shRNA-Syk by western blot. Notably, more MyD88 and iNOS were detected in Syk-knockdown microglia than in control cells (Figure 2D and E). Because tyrosines 519 and 520 are located in the activation loop of the Syk kinase domain and phosphorylation of Tyr519/520 is essential for Syk function, we measured Syk activation by detecting tyrosine phosphorylation at the residues 519/520. Pretreatment of BV-2 cells with PP1 resulted in inhibition of Syk activation and failed to attenuate MyD88 and iNOS expression (Figure 2D-F). In addition, both NF-κB- and iNOS-dependent gene reporter assays in BV-2 cells (data not shown) and primary microglia revealed that LLLT-mediated neuroprotective effect was dependent on Src/Syk-mediated down-regulation of TLRs signal pathway (Figure 2G). The western blot results clearly show that translocation of p65 into the BV-2 cells’ nucleus is reduced after LLLT treatment (Figure 2H-I). Furthermore, after LLLT treatment, the TNF-α production was increased drastically when Src or Syk was blocked (Figure 2J). These results suggested that Syk was functional in microglia and that LLLT-mediated anti-inflammatory signal in microglia was via Src/Syk.

### LLLT-mediated Syk activation does not require FAK

**Figure 3:**
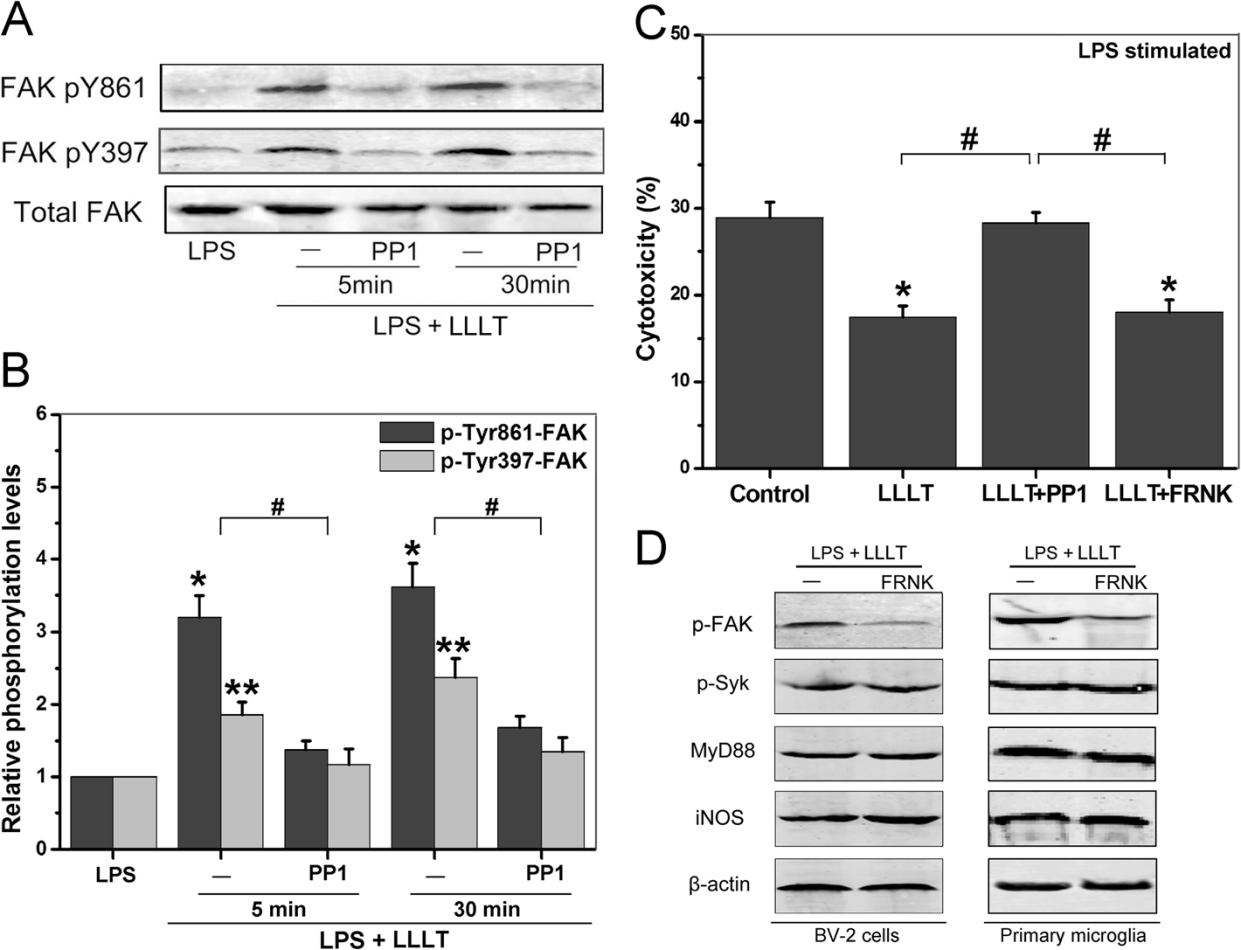
**Role of FAK in Syk-mediated neuroprotective effects under LLLT treatment.** (**A**) LPS-activated BV-2 cells pretreated with or without 10 μM PP1, followed by LLLT treatment. Western blot analysis of the treated cells was performed to detect phospho-Ty861-FAK, phospho-Tyr397-FAK, and total FAK at indicated time. (**B**) Quantitative analysis of the levels of phospho-Ty861-FAK, phospho-Tyr397-FAK, and total FAK in treated cells. Data represent mean ± SEM (n = 4; *P <0.05 and **P <0.05 *versus* corresponding control cells, #*P* <0.05 *versus* indicated cells). (**C**) BV-2 cells were transfected with GFP-FRNK (FRNK, an endogenous inhibitor of FAK). G418-resistant cells were collected for LPS (100 ng/ml) stimulation and LLLT (20 J/cm^2^) treatment. Immediately thereafter, SH-SY5Y cells were subjected to microglia cytotoxicity assay as previous described. Data represent mean ± SEM (n = 4; *P <0.05 *versus* control cells, #*P* <0.05 *versus* indicated cells). (**D**) Western blot analysis of the transfected cells received LPS stimulation and LLLT treatment to detect phospho-Tyr397-FAK, phospho-Tyr519/520-Syk, total MyD88, and total iNOS. Data were the representative graph (n = 4). iNOS, inducible nitric oxide synthase; LLLT, low-level laser therapy; LPS, lipopolysaccharide.

Src forms a complex with FAK which can phosphorylate other substrates and trigger multiple intracellular signaling pathways that induce different cellular responses [28]. We examined FAK phosphorylation on the Src-dependent tyrosine residue 861 (pY861), which is a well-characterized Src-dependent site, as well as the autophosphorylation site of FAK, pY397. Our results showed elevated levels of pY861-FAK in all LLLT-treated microglia except for the cells pretreated with PP1 (Figure 3A and B). However, cells expressing FAK related non-kinase (FRNK), an endogenous inhibitor of FAK, failed to show LLLT-induced Src-mediated neuroprotective effect on SH-SY5Y neuronal cells (Figure 3C). It appears that although FAK is activated under LLLT, it’s not involved in the downstream of Syk activation. This observation was further supported by western blot analysis in both BV-2 cells and primary microglia (Figure 3D).

### Effects of LLLT on LPS-activated microglial phagocytic activity

Given that phagocytosis is the process that results in the uptake of large particles (≥1 μm) by an actin-based mechanism, we ask whether FAK activation is due to microglial phagocytic activity after LLLT. Microglial phagocytosis was monitored by the internalization of fluorescent microspheres. Such microbeads have been used previously to investigate signaling in phagocytic cells [29].

**Figure 4:**
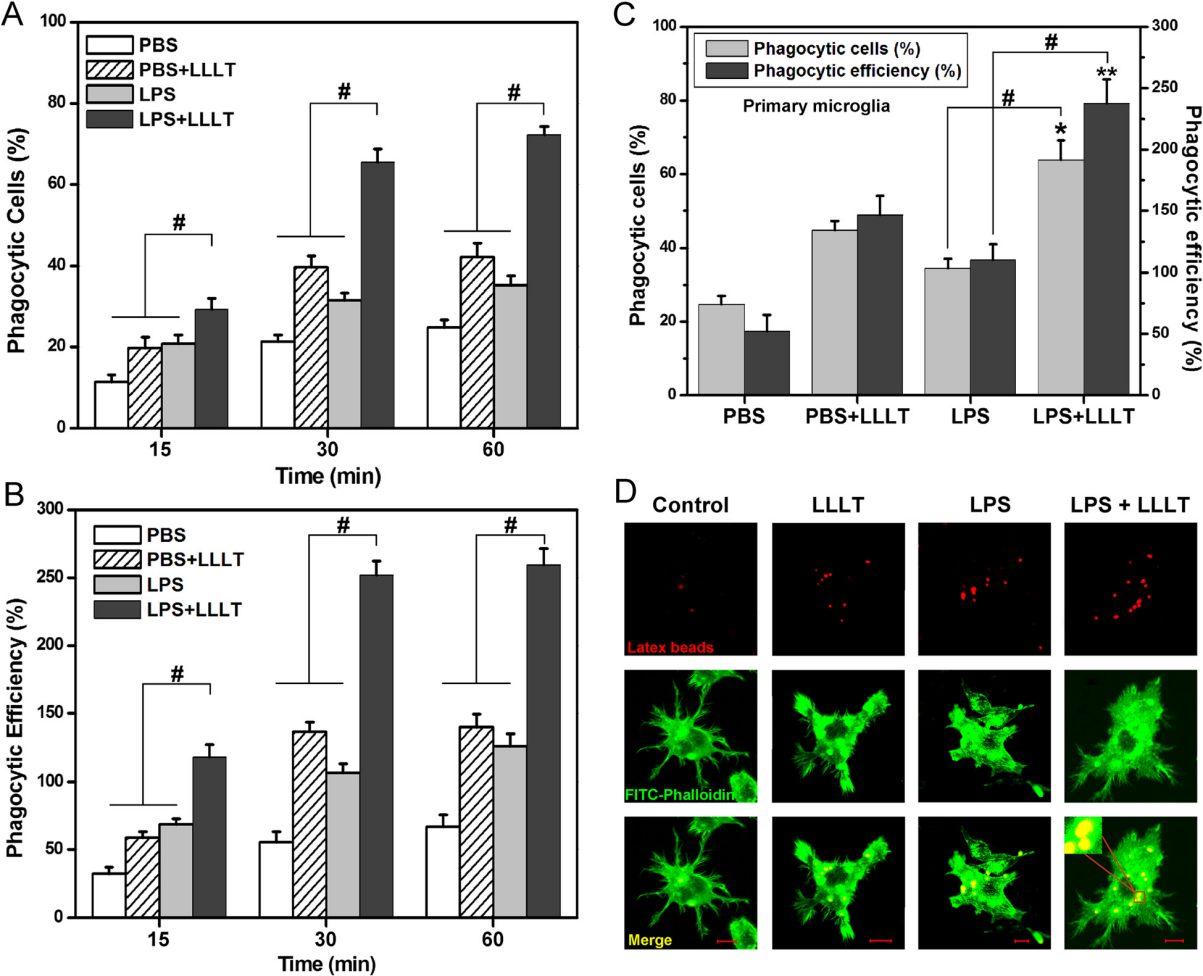
**Effects of LLLT on phagocytic function of activated microglial cells.** (A and B) BV-2 cells with or without LLLT (20 J/cm^2^) treatment incubated with microspheres for indicated time. (**A**) Quantification of LLLT-mediated microglial phagocytosis; (**B**) The number of microspheres taken up per BV-2 cell (n = 4; #*P* <0.05 *versus* indicated cells). (**C**) LLLT-mediated phagocytic response in primary microglia at 30 min. (n = 4; *P <0.05 and **P <0.05 *versus* corresponding control cells, #*P* <0.05 *versus* indicated cells). (**D**) Fluorescent images of the distinct microglial phagocytosis. Microglial BV-2 cells with or without LPS or LPS plus LLLT treatment. Immediately thereafter, fluorescent microspheres (red) were added for 30 min. Cells were then fixed with 4% paraformaldehyde and stained with FITC-labeled phalloidin to visualize F-actin (green) and confocal Z-stacks were acquired. Images shown are representative of approximately 100 cells. Bar = 10 μm. LLLT, low-level laser therapy; LPS, lipopolysaccharide.

LLLT enhanced microglial phagocytic function in a time-dependent manner (Figure 4A, B). For phagocytosis analysis, a control group (LPS treated group) subtraction was performed. Exposure of BV-2 cells to LLLT at a dose of 20 J/cm^2^ for 30 min produced the maximal phagocytic response, in which the relative percentage of phagocytized cells and that of phagocytic efficiency were 34.03 ± 5.1% and 145.48 ± 17.1%, respectively. The enhanced phagocytic activity was further demonstrated in primary microglia at 30 min after LLLT treatment (Figure 4C). Since actin assembly provides the driving force for particle engulfment by allowing the extension of membrane pseudopods that wrap the particle and eventually close to form a phagosome, we also observed F-actin recruitment and cupping around beads in BV-2 cells. LLLT markedly enhanced microglial phagocytosis, as shown in Figure 4D.

### Actin-based phagocytosis is a Rac1-dependent process

**Figure 5:**
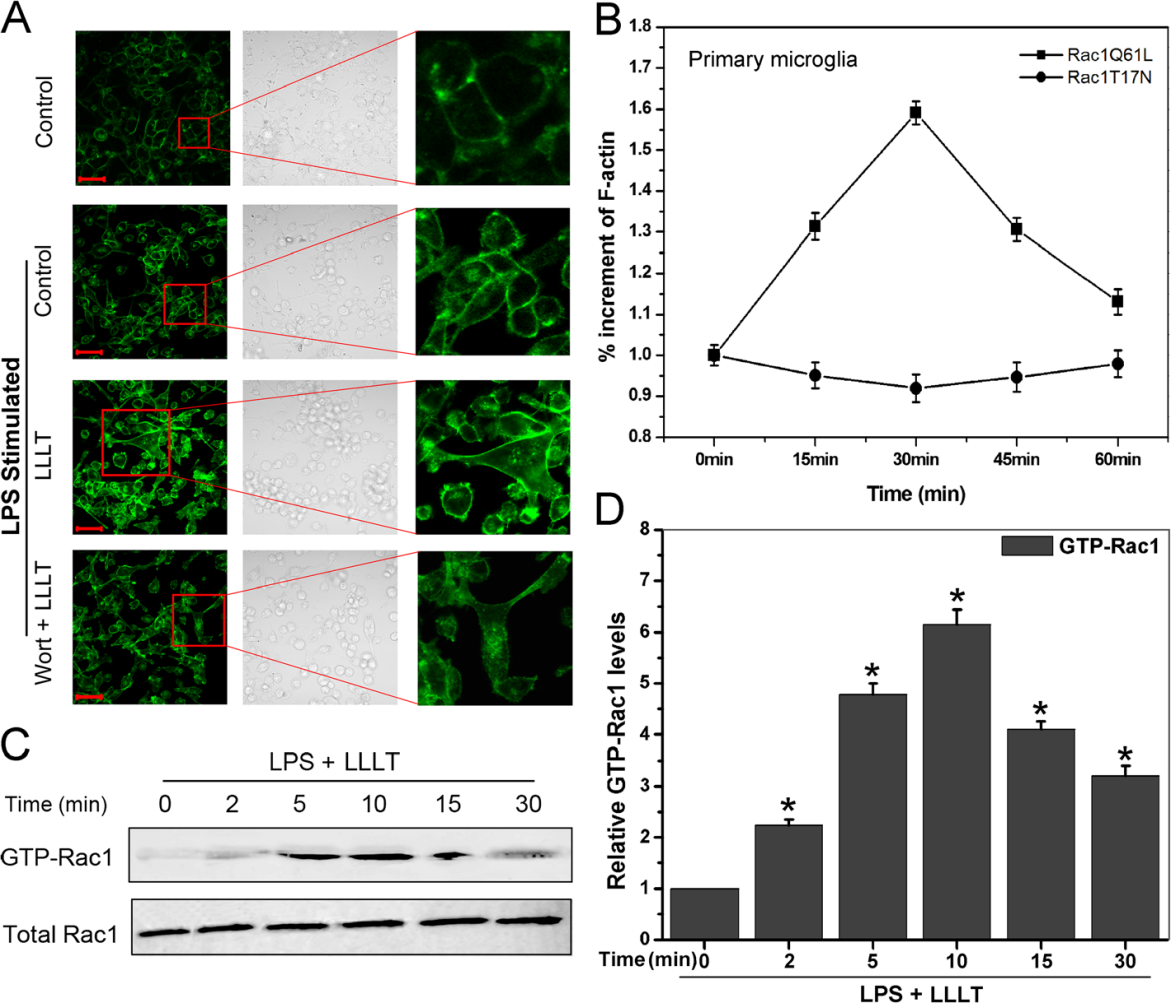
**LLLT-mediated microglial phagocytosis is a Rac-1-dependent actin-based process.** (**A**) LPS-activated BV-2 cells pretreated with or without wortmannin (100 nM) under LLLT treatment. 30 min after incubation, cells were fixed with 4% paraformaldehyde and stained with FITC-labeled phalloidin to visualize F-actin (green), and confocal Z-stacks were acquired. Images shown are representative of approximately 100 cells. Bar = 50 μm. (**B**) Primary microglia transfected with Rac1Q61L, Rac1T17N or wt-Rac1. G418-resistant cells were collected for further LPS stimulation. 30 min after LLLT (20 J/cm^2^) treatment, total F-actin was stained using FITC-phalloidin and measured using a 96-well plate reader. F-actin ratio was calculated as (F-actin in transfected cells - background)/(F-actin in wt-Rac1 transfected cells - background). Data represent mean ± SEM (n = 6). (**C**) Representative western blot analysis of LLLT-treated LPS-activated BV-2 cells to detect GTP-Rac1 and total Rac1. (**D**) Quantitative analysis of the levels of GTP-Rac1 and total Rac1 in treated cells. Data represent mean ± SEM (n = 4; *P <0.05 *versus* control cells). LLLT, low-level laser therapy; LPS, lipopolysaccharide.

Rac1 activation, by cycling between GDP-bound inactive and GTP-bound active conformations, has been shown to induce actin polymerization and is essential for cell phagocytosis [30-32]. We explored the role of Rac1 in LLLT-mediated microglial phagocytosis. As shown in Figure 5A, LLLT induced a significantly actin polymerization in BV-2 cells.

To study the effects of Rac1 activity on actin polymerization, we quantified F-actin content in LPS-activated primary microglia that were transfected with different Rac1 constructs under LLLT treatment. It’s clearly evident, as shown in Figure 5B, that a constitutively activated form of Rac1 (Rac1Q61L) induced a higher level of actin polymerization than cells transfected with wild-type Rac1, whereas a dominant negative form of Rac1 (Rac1T17N) markedly suppressed actin polymerization. In addition, the involvement of Rac1 activation after LLLT treatment was also confirmed by using a Raichu fluorescence resonance energy transfer (FRET)-based biosensor (data not shown).

In order to characterize the temporal effects of LLLT on Rac1 activation in BV-2 cells, we measured Rac1 activity from 2 min to 30 min. Rac1 was significantly activated within 2 min after LLLT and declined after 15 min (Figure 5C and D).

### LLLT-induced Rac1 activation is mediated by Src-dependent activation of PI3K/Akt signaling pathway

**Figure 6:**
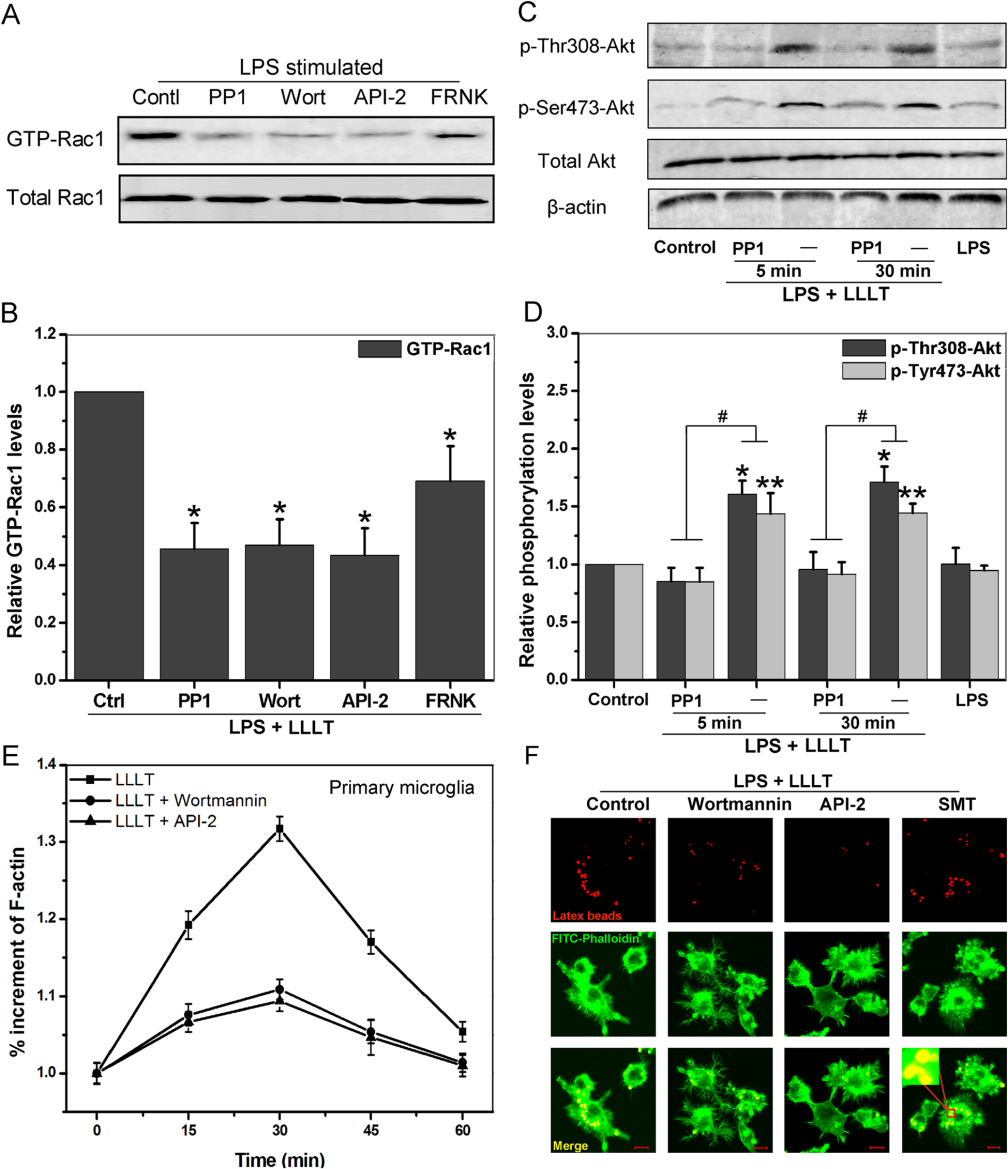
**Src regulates Rac1-dependent microglial phagocytosis through PI3K/Akt pathway.** (**A**) LPS-activated BV-2 cells pretreated with or without PP1 (10 μM), wortmannin (100 nM), or API-2 (5 μM), followed by LLLT (20 J/cm^2^) treatment. Cells stably transfected with GFP-FRNK were also collected for LPS stimulation and LLLT treatment. Western blot analysis of the treated cells was performed to detect GTP-Rac1 and total Rac1 at 10 min. Data were the representative graph. (**B**) Quantitative analysis of the levels of GTP-Rac1 and total Rac1 in treated cells. Data represent mean ± SEM (n = 4; *P <0.05 *versus* control cells,). (**C**) LPS-activated BV-2 cells pretreated with or without PP1, wortmannin, or API-2, followed by LLLT (20 J/cm^2^) treatment. The levels of phospho-Thr308-Akt, phospho-Ser473-Akt, and total Akt were detected with western blot analysis at indicated times. (**D**) Quantitative analysis of the levels of phospho-Thr308-Akt and phospho-Ser473-Akt in treated cells. (n = 4; *P <0.05 and **P <0.05 *versus* corresponding control cells, #*P* <0.05 *versus* indicated cells). (E and F) LPS-activated microglial cells pretreated with or without wortmannin, API-2, or SMT (100 μM) for 1 h, followed by LLLT (20 J/cm^2^) treatment. (**E**) Total F-actin in primary microglia was stained using FITC-phalloidin at the indicated times and measured as described in Materials and Methods. Data represent mean ± SEM (n = 6). (**F**) Fluorescent images of the distinct microglial phagocytosis in BV-2 cells. Merged beads (red) and F-actin (green) are displayed in yellow (bottom). Images shown are representative of approximately 100 cells. Bar = 10 μm. LLLT, low-level laser therapy; LPS, lipopolysaccharide.

Activation of PI3K/Akt signaling pathway has been correlated with macrophage phagocytosis [33]. To address whether Rac1 activation is mediated by PI3K/Akt signaling pathway, LPS-activated BV-2 cells were pretreated with wortmannin, which covalently inactivates the PI3K catalytic site, and with API-2, an Akt inhibitor. Both wortmannin and API-2 markedly reduced Rac1 activity after LLLT treatment (Figure 6A and B). Cells pretreated with PP1 reduced Rac1 activity, similar to wortmannin. Interestingly, cells transfected with FRNK, a naturally occurring inhibitor of FAK signaling, only caused partial inhibition of Rac1 activity. These results suggested that Src/FAK were involved in Rac1 activation and PI3K/Akt acted as an upstream event of Rac1.

We next investigated phosphorylation levels of Akt in BV-2 cells under LLLT (20 J/cm^2^) treatment. Date shown in Figure 6C and 6D revealed that LLLT caused significant induction of phospho-Thr308-Akt and phospho-Ser473-Akt, which were positively correlated with irradiation time, whereas total levels of Akt were unchanged. PP1 reduced the levels of both phospho-Thr308-Akt and phospho-Ser473-Akt. These results suggested that LLLT induced activation of the PI3K/Akt signaling pathway.

To determine whether the inhibition of PI3K/Akt signal in microglial cells prevents actin polymerization, we quantified F-actin content in primary microglia that were treated by LPS with or without wortmannin or API-2 after LLLT treatment. The presence of wortmannin or API-2 inhibited the F-actin accumulation (Figure 6E). PI3K inhibitor wortmannin or Akt inhibitor API-2 markedly suppressed BV-2 microglial phagocytosis (Figure 6F). Taken together, our results suggested that Src/PI3K/Akt pathway was required in the LLLT-induced Rac1 activation.

### Proinflammatory cytokines attenuate microglial phagocytosis

**Figure 7:**
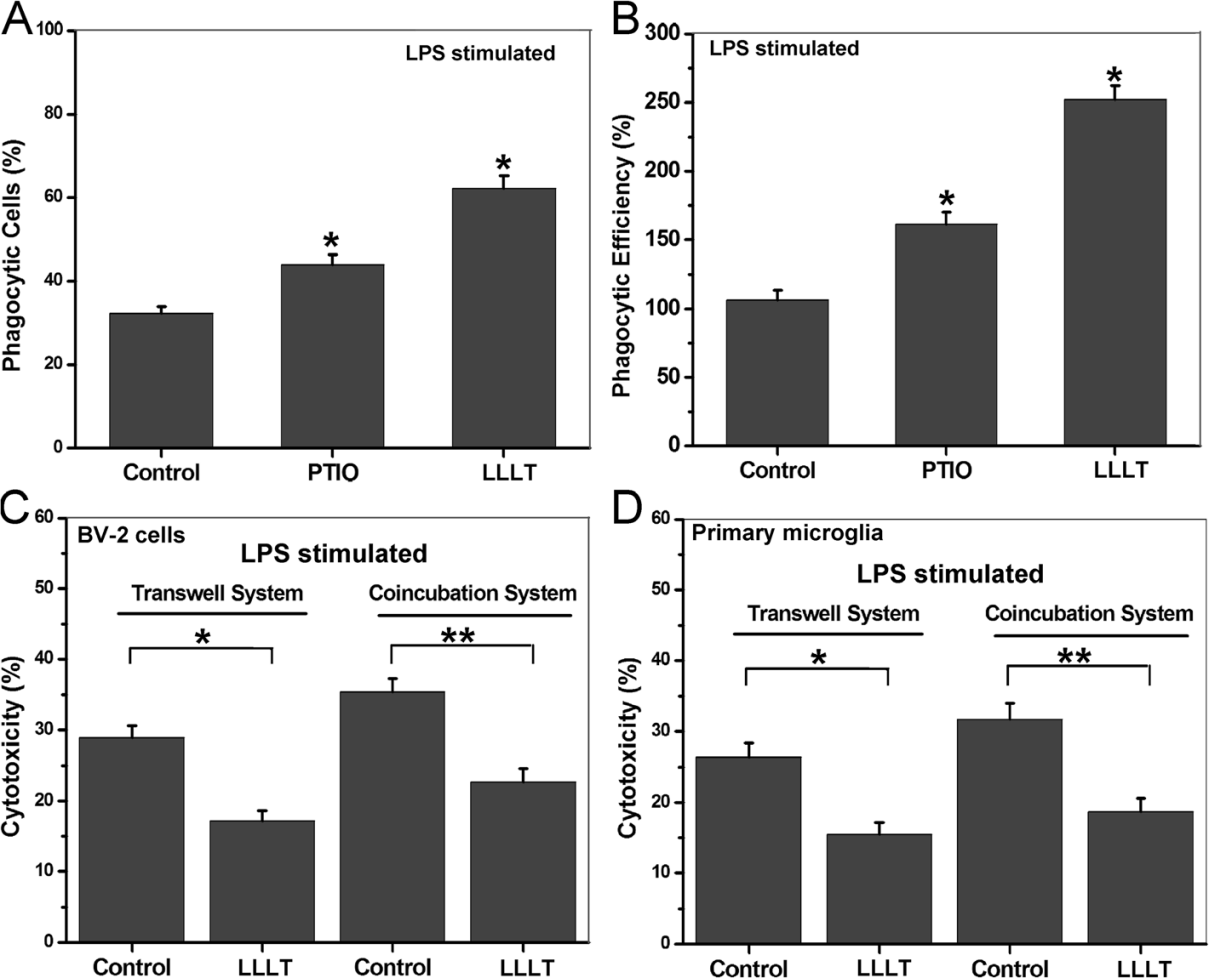
**Correlations between inflammatory mediators and the phagocytic response induced by LLLT in LPS-activated microglial.** (A and B) LPS-activated BV-2 cells pretreated with or without 200 μM carboxy-PTIO. Then the cells with or without LLLT (20 J/cm^2^) treatment were incubated with microspheres for 30 min. (**A**) Quantification of LLLT-mediated microglial phagocytosis. (**B**) Number of microspheres taken up per BV-2 cell. (n = 4; *P <0.05 *versus* control cells). (C and D) SH-SY5Y cells cultured with LPS-activated BV-2 cells or primary microglia (at an E:T ratio of 8:1) in a Transwell™ cell-culture system or directly in the mixed co-culture system for 24 h. After incubation, target cells that were doubly positive for CFSE and PI were analyzed by flow cytometry (n = 4; *P <0.05 and **P <0.05 *versus* indicated cells). CFSE, carboxyfluorescein diacetate succinimidyl ester; LLLT, low-level laser therapy; LPS, lipopolysaccharide; PI, propidium iodide.

Proinflammatory cytokines, such as NO and TNF-α, are capable of attenuating microglial phagocytosis by inducing actin depolymerization [34,35]. We further ask whether an LLLT-induced neuroprotective effect is positively correlated with microglial phagocytic activity. In BV-2 cells pretreated with SMT, an iNOS inhibitor, it’s clearly evident that inhibition of NO production significantly increased microglial phagocytic activity (Figure 6F). We also determined the percentage of phagocytized cells and that of phagocytic efficiency in BV-2 cells pretreated with Carboxy-PTIO, a NO scavenger, after LLLT treatment (Figure 7A, B). The results are consistent with the observations that are shown in Figure 6F.

Previous studies indicated that inhibition of microglial phagocytosis was sufficient to prevent inflammatory neuronal death [36]. To address whether LLLT-induced microglial phagocytic activity can trigger potent proinflammation and subsequently overrides its anti-inflammatory effects, microglial cytotoxicity assay was performed. PKH26-labled, LPS-activated BV-2 cells or primary microglia were treated by LLLT and then mix-cultured with PKH67-labled SH-SY5Y neuronal cells for 24 h. Compared with the results in Transwell™ cell-culture system, LLLT had a slight enhancement effect on neuron death (Figure 7C and D).

## Discussion

In the present study, we found a regulatory role of LLLT for microglial functions. Our major findings showed that LLLT significantly reduced LPS-activated microglia-induced neuronal cell death. In LPS-activated microglia-like BV-2 cells, LLLT attenuated inflammation cytokine TNF-α, decreased the production of NO, down-regulated overexpression of iNOS, and caused MyD88 degradation. Another important observation was that microglial phagocytosis was improved after LLLT treatment, characterized by an increase in Rac1 activity, actin polymerization and the ability of the microbeads to phagocytize. Moreover, we found that LLLT could induce the enhancement of microglial phagocytic activity by a Src-dependent PI3K/Akt pathway. Understanding the mechanism and functional significance of LLLT-induced microglia-mediated phagocytosis and neuroinflammation may lead to new neurotherapies.

AD is known to be associated with neuroinflammation and activated microglia. Uncontrolled and excessive activation of microglia is capable of releasing various potentially cytotoxic molecules such as NO, oxygen radicals, and proinflammatory cytokines such as TNF-α, IL-1β, and IL-6 [37,38]. Chronic neuroinflammation induced by excessive production of these neurotoxic molecules plays an important role in the degenerative process of AD patients. LPS acts as a potent stimulator of microglia and has been used to study the inflammatory process in the pathogenesis of AD and anti-inflammatory therapy for AD treatment. Evidence from some rat models showed that microglia were activated immediately after LPS injection. Significant elevations of cluster differentiation marker CD45, glial fibrillary acidic protein (GFAP), scavenger receptor A (SRA), and Fcγ receptor mRNA were seen after 24 h [39]. LPS-induced inflammation also exacerbates phospho-tau pathology in rTg4510 mice [40].

TLRs play a key role in microglia-mediated neuroinflammation. Previous studies revealed that integrin CD11b could be activated by TLR-triggered inside-out signaling, and then negatively regulated TLR-induced inflammatory response by promoting degradation of their adaptors [20]. Similar to the results of the present study, Mallard *et al.* demonstrated that microglial MyD88 signaling played an important role in regulating acute neuronal toxicity and MyD88 deficiency attenuated release of microglial proinflammatory cytokines following LPS exposure [41].

Much attention has been paid to therapeutic strategies aimed at controlling microglia-mediated neurotoxicity. LLLT is a non-thermal irradiation using light in visible to near infrared range which has been used clinically to accelerate wound healing and reduce pain and inflammation in a variety of pathologies [42,43]. Moreover, transcranial LLLT has shown good effects on treatment of stroke, traumatic brain injury, and neurodegenerative disease [44]. Although in many pre-clinical and clinical studies the 810-nm NIR light has been used for nerve repairs [45-47], the effects of laser irradiation with different wavelengths on microglial activation remain unclear. Studies had shown that the 632.8-nm laser had advantages over other wavelengths in treating neurological diseases [48,49]. Therefore, LLLT using the 632.8-nm laser may have high clinical relevance. Recently, it has been debated whether He-Ne laser light can activate a number of signaling pathways including MAPK/ERK, Src, Akt and RTK/PKCs signaling pathway [11,21,24,50].

We explored the role of Src activation involved in LPS-activated microglia after LLLT treatment. In our experiments, we demonstrated that LLLT triggered a significant activation of Src in LPS-activated BV-2 cells (Figure 1C). In addition, the activation of Syk triggered by LLLT was Src-dependent. LLLT-mediated Src/Syk activation could significantly decrease MyD88 and iNOS expression (Figure 2B). Blockade of Src activation or knockdown of Syk negatively affects neuronal survival under LLLT treatment (Figure 2A).

Excessive accumulation of NO has long been known to be toxic to neurons [51]. Oxygen-free radicals such as superoxide can react with NO to form deadly intermediates such as peroxynitrite. Our results indicated that LLLT could efficiently reduce LPS-activated microglia-induced iNOS (Figures 1D and 2C) by downregulating TLR-triggered proinflammation (Figure 2D - I).

How does LLLT activate Src? One of the most plausible explanations is that LLLT activates Src through reactive oxygen species (ROS). LLLT has been demonstrated to increase the level of intracellular ROS generation [52]. With LLLT treatment, light is absorbed by endogenous photosensitizers (porphyrins or cytochromes) that dominantly locate at plasma membrane, mitochondria or lysomes. The photosensitizers’ activation results in ROS (^1^O_2_, O_2_^-^, and H_2_O_2_) production [53]. Intracellular oxidants could mediate the activation of Src [54]. This hypothesis was also supported by our previous work [21]. Although there may be many other contributors responsible for LLLT-mediated neuroprotective effect, our experimental results suggest that Src and Syk are primary participants in downregulation of TLRs-triggered neuroinflammatory signaling pathway (Figures 2 and 3).

The deposition of Aβ in the extracellular space of the brain plays an important role in microglial activation in AD. Although the role of microglia-mediated inflammation in the pathogenesis of AD is obvious, microglia have been reported to mediate the clearance of Aβ through receptor-mediated phagocytosis, which could delay the progression of AD.

Recent studies suggested that Aβ oligomers could induce a potent inflammatory response and subsequently disturb microglial phagocytosis and clearance of Aβ fibrils, thereby contributing to an initial neurodegenerative characteristic of AD [26]. To address whether LLLT-mediated anti-inflammatory effects can improve microglial phagocytosis, we investigated the microglial phagocytic activity after LLLT treatment. Phagocytic activity of the LPS-activated microglia was markedly enhanced after LLLT treatment (Figures 3 and 6). Furthermore, LLLT could also activate the PI3K/Akt signal pathway (Figure 5), which was dependent on Src activation under LLLT treatment. Since phagocytosis is a Rac1-mediated actin-based process, our results demonstrated that not only the activity of Rac1 but also the F-actin accumulation were increased by PI3K/Akt after LLLT. A constitutively active form of Rac1 greatly increased F-actin polymerization, while a dominant negative form of Rac1 inhibited F-actin polymerization under LLLT treatment (Figure 5B). Thus, these results suggested that LLLT-induced Src activation could also improve microglial phagocytic activity by PI3K/Akt/Rac1 signal pathway.

Activation of the PI3K/Akt signaling pathway has been correlated with tumor metastasis and invasion [55]. Indeed, PI3K is a key signaling molecular for integrin activation and regulation of actin reorganization [56]. The nonreceptor tyrosine kinase FAK-Src complex can initiate a cascade of phosphorylation events to trigger multiple intracellular pathways, including MAPK/ERK and PI3K/Akt signaling [57,58]. In this study, using LPS-activated BV-2 cells, we found that LLLT-mediated anti-inflammatory effect did not require FAK. One of the most plausible explanations of the above results is that Syk activation does not require actin polymerization since it is unaffected by inhibitors such as cytochalasin D, whereas FAK activation requires actin polymerization [59,60]. Thus, activation of Syk, but not FAK, plays a key role in Src-mediated anti-inflammatory signal under LLLT. However, inhibition of FAK by transfecting BV-2 cells with FRNK, a naturally occurring inhibitor of FAK signaling, contributed to partial inhibition of microglia phagocytosis.

FAK translocation between cytosolic and membrane is highly regulated by many factors, including tyrosine phosphorylation and actin assembly [61]. Since p85 was associated with FAK to further activate Akt by binding to tyrosine phosphorylated residue 397 of FAK [62], this may explain why FAK could also be activated before actin polymerization and only had partial effects on Src-mediated microglial phagocytosis. Our results suggest that LLLT-induced phagocytic activity depends on Src-mediated PI3K/Akt signaling pathway, partially due to the phosphorylation of FAK.

Microglial activation is considered as a hallmark of AD. Alternatively, microglial activation is usually associated with marked increase in CD11b expression [63]. Integrin CD11b/CD18 (macrophage antigen complex 1, MAC1, also known as complement receptor 3, CR3) is essential for the phagocytosis of multiple compounds and mediates the activation of phagocytes in response to a diverse set of stimuli [64]. The MAC1 receptor is located on microglia, suggesting its role in neurodegeneration. In addition, previous reports indicated that MAC1 might be a key receptor for Aβ to activate microglia to produce extracellular superoxide, resulting in neurotoxicity [65]. Conversely, others indicated that ROS was a key player in microglial activation in which ROS increased microglial expression of CD11b via NO [66]. In fact, a recent study showed that activation of CD11b via inside-out signaling negatively regulated TLR-triggered proinflammatory response [20].

It is important to note that the role of microglial activation in AD is still debated, and a simplistic view of microglia as solely beneficial or detrimental cells does not reflect the complexity of microglial function [67]. Functionally, microglia react in diverse ways: they secrete inflammatory mediators, proteolytic enzymes or neurotrophic factors, and are also able to take up soluble and insoluble molecules. Hence, the ideal microglia-targeted AD treatment modalities should not only focus on microglial neurotoxic characteristics, but also on the phagocytic activity.

However, in this study, we used human neuroblastoma SH-SY5Y as the target neuronal cells to mimic responses of inflammation-mediated neurotoxicity. Given that microglia may have recognized markers of malignancy, our results need to be further confirmed using primary cortical neurons in the future studies.

Taken together, the current investigation demonstrates that LLLT can inhibit LPS-activated microglia-induced neurotoxicity and enhance its phagocytic activity through activation of non-receptor tyrosine kinase Src. Although cultured mouse microglia and its treatment with etiological reagents may not truly resemble microglia in the brain of patients, our results suggest that targeting Src may be an important step for the attenuation of microglial activation. Better understanding of the regulation mechanism of activated microglia may provide a therapeutic strategy to control the progression of neurodegenerative diseases.

## Abbreviations

Aβ: β-amyloid; AD: Alzheimer’s disease; CFSE: Carboxyfluorescein diacetate succinimidyl ester; CNS: Central nervous system; DMEM: Dulbecco’s modified Eagle’s medium; EGTA: Ethylene glycol tetraacetic acid; fAβ: Fibrillar β-amyloid; FEB: Fetal bovine serum; FRET: Fluorescence resonance energy transfer; iNOS: Inducible NO synthase; LLLT: Low-level laser therapy; LPS: Lipopolysaccharide; LSM: Laser scanning microscopy; NO: Nitric oxide; PD: Parkinson’s disease; PI: Propidium iodide; RNAi: RNA interference; ROS: Reactive oxygen species; SN: Substantia nigra; TLR: Toll-like receptor.

## Competing interests

The authors declare that they have no competing interests.

## Authors’ contributions

SS conceived the main idea of the study and performed the majority of the experiments and drafted the manuscript. FZ participated in the major experiments. SS and FZ analyzed the data. FZ and WRC contributed materials and reagents. FZ and WRC participated in its design and coordination and helped draft the manuscript. All authors reviewed and approved the final version of the manuscript.
